# Mixed-methods cross-sectional study of the prevention of vertical HIV transmission program users unaware of male partner’s HIV status, in six South African districts with a high antenatal HIV burden

**DOI:** 10.1186/s12889-023-16921-z

**Published:** 2023-10-12

**Authors:** Tshiamo M. Mmotsa, Vuyolwethu Magasana, Duduzile F. Nsibande, Mbongeleni Buthelezi, Reshmi Dassaye, Violeta J. Rodriguez, Deborah L. Jones, Ameena E. Goga, Nobubelo K. Ngandu

**Affiliations:** 1 HIV and other Infectious Diseases Research Unit, South African Medical Research Council, Cape Town, South Africa; 2 Department of Psychiatry and Behavioral Sciences, University of Miami Miller School of Medicine, Miami, FL USA; 3 Department of Psychology (Clinical/Community Division), University of Illinois at Urbana-Champaign, Champaign, IL, United States of America; 4 Department of Paediatrics and Child Health, University of Pretoria, Pretoria, South Africa

**Keywords:** HIV disclosure, Mother-to-child-transmission of HIV, Pregnant, Postpartum, Male partner

## Abstract

**Background:**

Elimination of vertical HIV Transmission (VHT) and maternal deaths are global health priorities. Male involvement is one of the most important factors that influences women’s decisions, including the uptake of Prevention of vertical HIV transmission (P-VHT). We sought to understand not knowing a male partner’s HIV status (MPHIVs) amongst women using services to prevent vertical HIV transmission in six South African districts with high antenatal HIV burden.

**Methods:**

A mixed-methods cross-sectional study was conducted in six South African districts, and data collected through face-to-face interviews with women and focus group discussions (FGDs) with women or male partners. The quantitative data were analyzed using STATA SE-17.0 and an inductive approach was used for qualitative data analysis.

**Results:**

Overall, 28.7% of women were unaware of their MPHIVs, while 25.3% and 46.0% knew the MPHIVs was positive or negative, respectively. In multivariable logistic regression, single marital status and unplanned pregnancy increased the odds of not knowing a MPHIVs while a woman’s disclosure of her HIV status to the male partner reduced the odds. FDGs highlighted complexities around MPHIVs disclosure, e.g., reluctance to test for HIV and potential interventions including healthcare worker (HCW) assisted HIV disclosure.

**Conclusion:**

User-informed interventions to address MPHIVs non-disclosure amongst women of child-bearing age, particularly those at risk of unstable sexual partners and unplanned pregnancies, should be strengthened.

**Supplementary Information:**

The online version contains supplementary material available at 10.1186/s12889-023-16921-z.

## Introduction

The Global Plan towards the elimination of vertical HIV transmission (VHT) and maternal deaths was introduced in 2011, in countries accounting for 90% of pregnant women living with HIV, including South Africa [1]. In 2020, 7.8 million people in South Africa were living with HIV and 24.7% of women of reproductive age were HIV-positive (15–49 years) [2]. Progress has been made towards the global targets in South Africa, with 97% of pregnant women with HIV initiated on antiretroviral therapy (ART) for Prevention of VHT (P-VHT) [2], the rate having reached 4% (2% at 6 weeks postpartum and 2% during the breastfeeding period) in 2020 [3]. However, the 2020 estimates indicate that South Africa is still one of the six countries accounting for more than 67% of HIV acquisition cases among children, most of which are through VHT [3].

Male involvement is one of the upstream factors indirectly influencing uptake of P-VHT programme activities amongst pregnant and postpartum women (PPW) and their infants. HIV disclosure between partners plays a particularly important role in the decisions that PPW make, especially regarding ART initiation and adherence [4]. A few studies have reported improved maternal and infant HIV clinical outcomes among women who had disclosed HIV status to male partners and knew their male partner’s HIV status (MPHIVs) [4–8]. According to van Lettow [4] and colleagues, postpartum women with partners who had not disclosed their HIV status to them were significantly less likely to be on ART or had suboptimal adherence to ART compared to postpartum women who knew their MPHIVs. In a study investigating mother-baby pairs, VHT was 4.6 times and 3.41 times significantly more likely to occur in mothers who had not disclosed their HIV status to a partner at 6 weeks and 6 months postpartum, respectively [5]. Additionally, a qualitative study conducted in Nigeria found that PPW who disclosed their HIV status to a male partner reported receiving encouragement and support for ART initiation and clinic visits from their partners [8].

The P-VHT programme has enabled increased uptake of HIV testing amongst women of reproductive age, reaching an antenatal HIV testing coverage of at least 90% in countries like South Africa and Lesotho in 2019 [9–12]. Although PPW have improved in testing for HIV, have generally improved in disclosing their HIV status to their partners, and factors associated with disclosure to male partners have been explored [13–15], a gap still remains in male partner disclosure to PPW. According to Manjate Cuco et al. [16], male partners of PPW were not interested in HIV prevention nor in knowing their HIV status and thus low uptake of HIV testing services (HTSs). A study in South Africa among HIV-positive heterosexual men and women found that 45% of women did not know their MPHIVs compared to only 13% of men not knowing their female partner’s HIV status [17]. Another study conducted in Ethiopia found that differences in knowledge of a partner’s HIV status between HIV-positive heterosexual men and women were not as significant, 22% of women versus 19.8% of men did not know their partner’s HIV status [18]. Various studies in Nigeria, Kenya and South Africa found that the prevalence of not knowing a MPHIVs amongst PPW ranged from 32.4 to 39.4% [19–21]. These findings are alarming given long-standing reports of low condom use amongst male partners of pregnant women and women of reproductive age in these settings [3, 22–24], leaving them at risk of co-infection with HIV and other sexually transmitted infections.

It is important to understand patterns of male partner disclosure to their pregnant and postpartum female partners to improve progress towards the P-VHT global targets. This study aimed to explore the prevalence of not knowing a MPHIVs and its associated factors amongst postpartum women in six districts with a high burden of antenatal HIV in South Africa.

## Methods

### Study design and setting

A mixed methods cross-sectional study design was used combining qualitative primary data and quantitative secondary data analysis of the same study. The primary study was a cross-sectional process evaluation of P-VHT Option B + implementation conducted in six South African districts from February 2018 through to June 2018, to assess program performance two years after the national rollout. Study districts were selected from 25 districts with the highest infant and antenatal HIV prevalence during the study planning period [11] and were; a peri-urban district- OR Tambo in Eastern Cape province (Periurban_EC), two urban metros- Ekurhuleni in Gauteng province (Urban_GP) and eThekwini in KwaZulu Natal province (Urban_KZN), two rural districts - Greater Sekhukhune in Limpopo (Rural_LP) and Bojanala in North West province (Rural_NW), and one peri-urban district with a large rural setting- Ehlanzeni in Mpumalanga province (Rural/periurban_MP).

### Quantitative data collection and analysis

The quantitative data collection questionnaires were completed by interviewing participants at managerial/policy maker level, service delivery level (with healthcare workers (HCWs)) user level (maternal interviews) and assessing facility registers/documentation. Quantitative secondary data from maternal interviews conducted in all six districts were used in this manuscript.

#### Sample size

Sample sizes for the quantitative component in the primary study were determined to estimate district-level maternal data for key P-VHT indicators and are described in detail in Ngandu et al., 2021 [25]. Briefly, four groups of mother-infant pairs were enrolled in each district to broadly represent the P-VHT program and comprised: HIV-positive mothers with infants aged 4–14 weeks (early postpartum HIV-exposed group) or 6–12 months (mid postpartum HIV-exposed group); HIV-negative mothers with infants aged 4–14 weeks (early postpartum unexposed group) or 6–12 months (mid postpartum unexposed group). Non-probability convenience sampling was used. The minimum target sample size was 120 mother-baby pairs per stratum per district, determined using an absolute precision of 10% and a design effect of 2, to report district-level estimates of primary outcomes. Enrolment took place in public primary healthcare facilities and targeted women who had been attending the selected facilities for at least six months for maternity or other mother and child health services.

#### Variables

The primary outcome variable was maternal participant’s knowledge of MPHIVs presented as a categorical variable grouped into ‘know MPHIVs is negative’, ‘know MPHIVs is positive’ and ‘don’t know MPHIVs’. Out of all the exposure variables that were available in the dataset, the authors considered the basic socio-demographic factors and P-VHT or healthcare-related factors that they deemed would be influenced by non-disclosure of MPHIVs. Available sociodemographic and healthcare related factors included; categorical variables: maternal age (13–24, 25–34 and 35–49 years old), parity (first child, 2–3 children, 4 or more children), ART initiation timing (before most recent pregnancy, during recent pregnancy or after delivery), infant testing coverage (yes, no, don’t know); yes/no binary variables: unplanned pregnancy, ever breastfed, woman disclosed HIV status to partner, experienced clinic visit barriers, experienced challenges with adherence to ART; and other binary variables: highest education (high school & higher versus primary or none), marital status (married/cohabiting versus single), timing of first HIV-positive result (before most recent pregnancy versus at ANC first visit or after) and socio-economic status (SES, calculated from household assets and source of income using principal component analyses and classified into high and low SES). Variables which defined the study strata were also included and these were: district (categorical), maternal HIV status (binary) and postnatal period (a binary of the infant age-groups).

#### Statistical analysis

The study was designed to present results at district levels, hence all proportions and point estimates were weighted to account for sample size realization within each stratum and for the sampling frame of facilities within each district. A chi-squared test was used to describe the distribution of MPHIVs against exposure variables. A polytomous multivariable logistic regression model, using the mlogit function in STATA, was used to determine associations between ‘knowing/not knowing a MPHIVs and exposure variable in a two-step process. In the first step, univariate mlogit tests were conducted and exposure variables with an overall p-value < 0.2 were shortlisted. In the second step, the shortlisted variables were included in a multivariable (adjusted) model to report adjusted relative risk ratios (aRRR). The regression analyses were conducted for the entire sample and for the sub-sample of HIV-exposed infants (i.e., HIV-positive maternal participants).

All analyses were performed in STATA SE-17 and a 5% (p value < 0.05) significance level was used.

### Qualitative data collection and analysis

#### Sampling technique

Focus group discussions (FGDs) were used to collect experiences and perceptions around HIV disclosure by male partners from the following groups of participants: four groups of postpartum women (HIV-positive and older (aged >24 years), HIV-negative and older (aged >24 years), HIV-positive adolescent girls and young women aged 24 years and younger (AGYW), HIV negative AGYW), pregnant women regardless of age and HIV status and men. Six to ten participants were targeted per group (to meet the requirement to conduct a FGD) in each of the six districts. The targeted participants were purposively recruited at the healthcare facilities included in the study. Participants who showed interest in the study were enrolled after providing signed informed consent. Nineteen FGDs were conducted in the final achieved sample: four with older HIV-positive postpartum women (in Periurban_EC, Urban_GP, Urban_KZN, and Rural/periurban_MP); six with pregnant women regardless of HIV status and age (one per district- Peri-urban_EC, Urban_GP, Urban_KZN, and Rural/peri-urban_MP; Rural NW, Rural LP); two with males (Urban_KZN, and Rural/periurban_MP); six with HIV-negative older postpartum women (one per district- Peri-urban_EC, Urban_GP, Urban_KZN, and Rural/peri-urban_MP; Rural NW, Rural LP)); and one with HIV-negative AGYW (Urban_KZN).

#### Conduct of FGDs

The Consolidated criteria for Reporting Qualitative (COREQ) research checklist was used as a reference guide for the qualitative component [26] (Additional file 1). Two female researchers trained in qualitative data collection and thematic framework analytic methods conducted FGDs between 19 April 2018–14 May 2018 and 6–13 November 2018. Both were experienced project leaders, with Master in Public Health (MPH) Degrees, 18 years’ and 14 years’ experience in qualitative data collection and with expertise in conducting maternal and child health (MCH) research projects, including P-VHT. They had no prior contact or relationship with any of the research participants before the study interviews. Women and male partners visiting primary healthcare facilities for MCH services were purposively recruited from the waiting room and were offered enrolment into the study while waiting for care, if they met inclusion criteria (Additional file 2). FGDs were held on the same day of the visit using interview guides with prompts. FGDs were audio-recorded and lasted approximately 45 min. Interviews were conducted in the participants’ local languages (isiZulu, isiXhosa, Seswati, SeSotho, Setswana and Sepedi) and were transcribed in English. One researcher conducted a quality check for all the transcribed interviews and made corrections as needed by listening to the audio tapes in comparison with the transcript.

Researchers took field notes during the sessions and discussed data saturation at the end of the clinic visit.

#### Data synthesis and analysis

An inductive approach was used to understand the participants’ perspectives. In this approach, researchers generate an explanation of the process, action, or interaction shaped by the views of many participants. The inductive approach allows research findings to emerge from the frequent, dominant or significant themes inherent in raw data, without the restraints imposed by structured methodologies. Raw text data is then condensed into a brief summary format; clear and justifiable links between the research objectives are developed into a theory/model [27, 28].

The first phase of data analysis involved the two qualitative researchers sampling and reading the same 10 English transcripts to familiarise themselves with the data. The two researchers had a meeting where they discussed and identified themes that emerged from the transcripts. Following the discussion on the emerging themes they developed an excel spreadsheet and assigned codes independently using a thematic framework analysis [29]. Thematic framework analysis is an interpretive process that is often used to identify patterns from the data systematically. It is a suitable analysis method for qualitative policy evaluation research [30]. The two qualitative researchers coded all the FGDs independently and met weekly to discuss new emerging themes, data interpretation and concluded their findings.

For transparency and confirmability, the second phase of the analysis involved a secondary analyst. For reliability and credibility, the last phase of the analysis involved the study team (of which some became the authors of this manuscript) where initial/preliminary findings were presented using thematic analysis on excel spreadsheets, followed by identification of summaries and quotes that were relevant for this manuscript. Leading authors including two qualitative and two quantitative researchers and one study support team member had weekly discussions on the study findings and all the input from the leading authors was taken into consideration in finalising the analysis. Direct quotes were presented to support the researchers’ conclusions.

## Results

### Observations from the quantitative data

#### Population characteristics

A total of 2072 postpartum women were enrolled across the six districts, with 853 (48.8%) HIV-positive women and 1219 (51.2%) HIV-negative women (Table 1). Nearly half of the women were aged between 25 and 34 years (47.9%) and just over half (51.9%) had between two and three children, were from low SES, initiated ART before current pregnancy and reported not experiencing challenges with ART adherence. Regardless of HIV status, most participants reported that they were single (69.5%), had completed at least one year of high school education (91.7%), were breastfeeding (79.4%), and had disclosed their HIV status to their male partner (88.0%). Additionally, of the 853 HIV-positive women, 65.2% and 56.8% were diagnosed with HIV and initiated on ART before the current pregnancy, respectively.

**Table 1:** Characteristics of the study sample (N = 2072)

Factor	*n*	weighted %
*Total N*	*2072*	*100*
**District**		
Periurban_EC	321	20.3
Urban_GP	273	13.5
Urban_KZN	385	19.3
Rural/periurban_MP	355	17.0
Rural_LP	399	13.1
Rural_NW	339	16.8
**Postnatal period**		
4–14 weeks	921	50.6
6–12 months	1151	49.4
**Maternal HIV status**		
Negative	1219	51.2
Positive	853	48.8
**Maternal age**		
35–49 years	357	18.2
25–34 years	975	47.9
13–24 years	740	33.5
Highest education*		
High School & higher	1887	91.7
Primary or none	181	08.3
Marital status*		
Married/cohabiting	625	30.2
Single	1444	69.5
Parity*		
First child	709	33.3
2–3 children	1070	51.9
4–8 children	288	14.5
Unplanned pregnancy*		
No	986	45.3
Yes	1082	54.5
**Ever breastfed (other = No)**		
No	382	20.6
Yes	1690	79.4
**SES**		
High	1027	48.3
Low	1045	51.7
Woman disclosed HIV status to partner*		
No	235	12.0
Yes	1835	88.0
**Infant testing coverage**		
No	432	18.8
Yes	1512	73.7
No answer/Don’t know	128	07.5
**Factors collected from HIV-positive women only**		
***Total N***	***853***	***100***
**Timing of First HIV-positive result**		
Before most recent pregnancy	556	65.2
At ANC first visit or after	297	34.8
**ART initiation timing**		
Before most recent pregnancy	490	56.8
During recent pregnancy or after delivery	320	38.1
No answer/Don’t know	43	05.1
**Experience challenges with ART adherence** ^**a**^		
No	473	54.1
Yes	380	45.9
**Experience clinic visit barriers**		
Yes	592	68.6
No	261	31.4

Periurban_EC = OR Tambo District in Eastern Cape Province; Urban_GP = Ekurhuleni District in Gauteng Province; Urban_KZN = eThekwini District in KwaZulu-Natal Province; Rural/periurban_MP = Ehlanzeni District in Mpumalanga Province; Rural_LP = Greater Sekhukhune District in Limpopo Province; Rural_NW = Bojanala District in North West Province; SES = socio-economic status; ART = antiretroviral therapy; N = total number of clients interviewed. *variable has some missing responses totaling less than 2%

#### Prevalence of not knowing a MPHIVs

The prevalence of the outcome and its distribution by exposure variable is presented in Table 2. The prevalence of not knowing a MPHIVs amongst all enrolled women was 28.7%, while 46.0% and 25.3% of the women knew their male partner was HIV-negative or HIV-positive, respectively. When comparing by women’s HIV status, knowledge of a concordant MPHIVs appeared to be significantly higher in each group compared to the other (knowledge of concordant MPHIVs was 71.9% and 47.8% amongst HIV-negative and HIV-positive women, respectively (p-value < 0.0001), while the prevalence of not knowing a MPHIVs ranged between 26.4 and 30.9% amongst HIV-negative and HIV-positive women. However, a higher prevalence of not knowing a MPHIVs was observed amongst women who: had not disclosed their own HIV status to the male partner, were single, reported unplanned pregnancy for the index child, were from low SES backgrounds, and were enrolled from Periurban EC and rural LP districts (all p-values < 0.01). Although the point prevalence of not knowing a MPHIVs did not differ by women’s age, a significantly lower proportion of younger women knew their male partner was HIV-positive (15.1% [95% confidence intervals (CI) 11.6, 19.5] among 13–24 years old group compared to over 29.0% in older women, p-value < 0.0001). Similar to younger women, low prevalence of knowing a HIV-positive MPHIVs was observed amongst women who: only had one child, practiced breastfeeding, reported facing barriers to clinic attendance or had not experienced challenges with ART adherence. Although the prevalence of not knowing a MPHIVs did not differ by timing of ART initiation relative to pregnancy, amongst HIV-positive women, the majority of those who had missing information on their ART initiation status had reported to be in an HIV discordant relationship. More HIV-positive women who reported facing challenges with ART adherence were in known HIV concordant sexual relationships in comparison to those who reported not facing these challenges (48.0% [41.3, 54.8] versus 18.3 [15.8, 21.2], p-value < 0.0001).

**Table 2:** Characteristics of the study sample by knowledge of male partner’s HIV status (MPHIVs)

		Know partner is HIV negative	Know partner is HIV positive	Don’t know partner’s HIV status	
	n	Weighted % [95%CI]	Weighted % [95%CI]	Weighted% [95%CI]	p-value
***TOTAL N***	***2072***	46.0 [43.0, 48.9]	25.3 [22.9, 28.0]	28.7 [26.1, 31.5]	
**Factor**					
**District**					0.0093^*^
Periurban_EC	321	40.8 [33.2, 48.8]	20.2 [14.0, 28.2]	39.1 [31.1, 47.6]	
Urban_GP	273	54.5 [46.2, 62.5]	25.0 [18.1, 33.4]	20.6 [15.4, 27.0]	
Urban_KZN	385	44.3 [34.4, 54.6]	31.2 [23.5, 40.0]	24.6 [19.1, 31.0]	
Rural/periurban_MP	355	49.1 [41.2, 57.0]	28.5 [22.0, 36.1]	22.4 [17.3, 28.5]	
Rural_LP	399	48.2 [40.0, 56.5]	17.8 [12.1, 25.6]	33.9 [28.8, 39.5]	
Rural_NW	339	46.0 [43.0, 49.0]	25.3 [22.9, 28.0]	28.7 [26.1, 31.5]	
**Postnatal period**					0.908
4–14 weeks	921	45.5 [41.4, 49.8]	25.2 [21.6, 29.2]	29.3 [25.3, 33.7]	
6–12 months	1151	46.4 [42.3, 50.5]	25.5 [22.4, 28.9]	28.1 [24.9, 31.5]	
**Maternal HIV status**					< 0.0001^*^
Negative	1219	71.9 [68.6, 75.0]	01.7 [01.1, 02.7]	26.4 [23.3, 29.7]	
Positive	853	21.3 [17.9, 25.1]	47.8 [43.6, 52.0]	30.9 [26.9, 35.3]	
**Maternal age**					< 0.0001^*^
35–49 years	357	39.3 [32.9, 46.1]	34.0 [28.1, 40.4]	26.7 [21.1, 33.1]	
25–34 years	975	41.6 [37.5, 45.8]	29.2 [25.8, 32.9]	29.2 [25.2, 33.5]	
13–24 years	740	56.2 [51.0, 61.3]	15.1 [11.6, 19.5]	28.6 [24.5, 33.2]	
**Highest education**					0.057
High School & higher	1887	45.8 [43.8, 49.8]	25.3 [22.8, 28.0]	27.9 [25.2, 30.8]	
Primary or none	181	37.5 [29.1, 46.7]	25.5 [19.2, 33.1]	37.0 [29.0, 45.8]	
**Marital status**					< 0.0001^*^
Married/cohabiting	625	53.5 [48.1, 58.8]	29.1 [24.5, 34.2]	17.4 [13.9, 21.6]	
Single	1444	42.8 [39.1, 46.6]	23.8 [20.5, 27.3]	33.4 [30.3, 36.7]	
**Parity**					< 0.0001^*^
First child	709	55.5 [50.3, 60.5]	17.3 [13.3, 22.1]	27.2 [23.2, 31.7]	
2–3 children	1070	40.9 [37.2, 44.7]	28.9 [25.6, 32.5]	30.2 [26.3, 34.3]	
4–8 children	288	42.2 [34.6, 50.2]	30.3 [23.6, 38.0]	27.5 [26.2, 31.6]	
**Unplanned pregnancy**					< 0.0001^*^
No	986	50.3 [46.4, 54.2]	27.9 [24.3, 31.7]	21.9 [19.0, 25.2]	
Yes	1082	42.5 [38.5, 46.6]	23.1 [19.9, 26.6]	34.5 [30.6, 38.6]	
**Ever breastfed (other = No)**					< 0.0001^*^
No	382	33.5 [27.5, 40.1]	39.1 [32.1, 46.5]	27.5 [21.6,34.2]	
Yes	1690	49.2 [45.9, 52.5]	21.8 [19.3, 24,5]	29.0 [26.2, 32.1]	
**SES distribution**					0,001^*^
High	1027	49.9 [45.1, 54.7]	27.1 [22.9, 31.7]	23.0 [19.9, 26.6]	
Low	1045	42.3 [38.4, 46.2]	23.7 [20.4, 27.4]	34.0 [30.2, 38.0]	
**Woman disclosed HIV status to partner**					< 0.0001^*^
No	235	16.6 [11.8, 22.9]	05.7 [03.0, 10.5]	77.7 [70.9, 83.3]	
Yes	1835	49.9 [46.8, 53.1]	28.0 [25.3, 30.9]	22.0 [19.4, 24.9]	
**Infant testing coverage**					< 0.0001^*^
No	432	63.9 [57.5, 69.9]	04.6 [02.6, 08.1]	31.4 [25.7, 37.8]	
Yes	1512	42.6 [39.3, 46.0]	30.5 [27.5, 33.6]	26.9 [24.1, 30.0]	
No answer/Don’t know	128	33.6 [22.4, 47.0]	26.9 [18.4, 37.4]	39.5 [28.5, 51.7]	
**Factors collected from HIV-positive women only**					
***Total N***	***853***	21.3 [17.8, 25.1]	47.8 [43.6,52.0]	30.9 [26.9,35.3]	
**Timing of First HIV positive result**					0.073
Before most recent pregnancy	556	23.0 [18.8, 27.9]	49.1 [44.3, 53.9]	27.9 [23.5, 32.8]	
At ANC first visit or after	297	18.0 [13.2, 24.0]	45.4 [38.4, 52.6]	36.6 [29.6, 44.2]	
**ART initiation timing**					< 0.0001^*^
Before most recent pregnancy	490	21.8 [17.8, 26.5]	51.4 [46.3, 56.6]	26.7 [22.1, 32.0]	
During recent pregnancy or after delivery	320	20.6 [15.3, 27.2]	42.0 [35.3, 49.0]	37.4 [20.9, 44.4]	
No answer/Don’t know	43	69.3 [65.9, 72.5]	04.2 [02.9, 06.0]	26.5 [23.5, 29.7]	
**Experience challenges with ART adherence**					< 0.0001^*^
No	473	53.3 [50.0, 56.5]	18.3 [15.8, 21.2]	28.8 [25.6, 31.3]	
Yes	380	22.2 [16.9, 28.6]	48.0 [41.3, 54.8]	29.8 [23.4, 37.1]	
**Experience clinic visit barriers**					0.345
No	261	18.5 [13.1, 25.4]	46.6 [39.2, 54.1]	34.9 [27.7, 42.9]	
Yes	592	22.5 [18.4, 27.2]	48.4 [43.2, 53.5]	29.1 [24.6, 34.1]	

Periurban_EC = OR Tambo District in Eastern Cape Province; Urban_GP = Ekurhuleni District in Gauteng Province; Urban_KZN = eThekwini District in KwaZulu-Natal Province; Rural/periurban_MP = Ehlanzeni District in Mpumalanga Province; Rural_LP = Greater Sekhukhune District in Limpopo Province; Rural_NW = Bojanala District in North West Province; SES = socio-economic status; ART = antiretroviral therapy, N = total number of clients interviewed. *significant chi-squared p-values at p < 0.05

#### Factors associated with not knowing a MPHIVs

In the adjusted multivariable analysis age, education, parity, breastfeeding and SES distribution were not significantly associated with not knowing a MPHIVs amongst all the women in the sample regardless of HIV status. However, women who were single and those who had an unplanned pregnancy were significantly more likely to not know their MPHIVs, adjusted relative risk ratio (aRRR) 1.9; 95%CI 1.0-3.5; *p* = 0.042 and aRRR 2.2; 95%CI 1.2–3.9; *p* = 0.007, respectively. Additionally, women who disclosed their HIV status to a partner were significantly less likely to not know their MPHIVs (aRRR 0.1; 95%CI 0.1–0.3; *p* < 0.0001) (Table 3).

**Table 3:** Factors associated with not knowing male partner’s HIV status amongst all women

*base sub-group = “know partner is HIV-negative"*	Know partner is HIV positive			Don’t know partner’s HIV status		
	**aRRR**	**95% CI**	**p-value**	**aRRR**	**95% CI**	**p-value**
**Maternal age**						
35–49 years	***ref.***			***ref.***		
25–34 years	1.1	0.6–2.0	0.640	1.2	0.6–2.4	0.581
13–24 years	1.3	0.6–3.2	0.506	1.3	0.6–3.2	0.523
**Highest education**						
High School & higher	***ref.***			***ref.***		
Primary or none	1.1	0.6–2.5	0.674	2.1	0.9–4.9	0.101
**Marital status**						
Married/cohabiting	***ref.***			***ref.***		
Single	1.0	0.6–1.9	0.888	1.9	1.0–3.5	0.042^*^
**Parity**						
First child	***ref.***			***ref.***		
2–3 children	0.9	0.5–1.8	0.837	1.5	0.8–2.6	0.203
4–8 children	0.7	0.3–1.8	0.475	0.9	0.4–2.1	0.788
**Unplanned pregnancy**						
No	***ref.***			***ref.***		
Yes	1.1	0.7–1.7	0.792	2.2	1.2–3.9	0.007^*^
**Ever breastfed (other = No)**						
No	***ref.***			***ref.***		
Yes	1.0	0.6–1.7	0.957	1.1	0.6–1.9	0.828
**SES distribution**						
High	***ref.***			***ref.***		
Low	0.7	0.4–1.2	0.203	1.1	0.7–1.9	0.682
**Woman disclosed HIV status to partner**						
No	***ref.***			***ref.***		
Yes	2.2	0.8–5.9	0.124	0.1	0.1–0.3	< 0.0001^*^

aRRR = adjusted relative risk ratio; CI = confidence interval; SES = socio-economic status; *significant p-values at p < 0.05

**Table 4:** Factors associated with not knowing male partner’s HIV status amongst HIV-positive women only

*base sub-group = “know partner is HIV-negative"*	Know partner is HIV-positive			Don’t know partner’s HIV status		
	**aRRR**	**95% CI**	**p-value**	**aRRR**	**95% CI**	**p-value**
**District**						
Periurban_EC	***ref.***			***ref.***		
Urban_GP	0.6	0.3–1.5	0.304	0.5	0.2–1.3	0.135
Urban_KZN	1.3	0.7–2.4	0.487	0.6	0.2–1.5	0.249
Rural/periurban_MP	1.8	0.8–3.9	0.152	0.9	0.3–2.3	0.828
Rural_LP	0.8	0.3–1.7	0.513	1.3	0.5–3.1	0.610
Rural_NW	1.8	0.8–3.8	0.157	0.9	0.3–2.8	0.874
**Maternal age**						
35–49 years	***ref.***			***ref.***		
25–34 years	1.0	0.5–1.8	0.958	1.2	0.6–2.4	0.566
13–24 years	1.1	0.4–2.5	0.879	1.4	0.6–3.5	0.445
**Highest education**						
High School & higher	***ref.***			***ref.***		
Primary or none	1.0	0.5–2.2	0.864	2.2	0.9–5.2	0.076
**Marital status**						
Married/cohabiting	***ref.***			***ref.***		
Single	1.0	0.5–1.8	0.933	2.0	1.0–3.9	0.038^*^
**Parity**						
First child	***ref.***			***ref.***		
2–3 children	0.9	0.5–1.7	0.712	1.4	0.8–2.6	0.245
4–8 children	0.7	0.3–1.7	0.456	0.8	0.4–2.0	0.708
**Unplanned pregnancy**						
No	***ref.***			***ref.***		
Yes	1.1	0.7–1.8	0.716	2.4	1.4–4.3	0.003^*^
**SES distribution**						
High	***ref.***			***ref.***		
Low	0.6	0.35–1.1	0.118	0.9	0.5–1.5	0.618
**Woman disclosed HIV status to partner**						
No	***ref.***			***ref.***		
Yes	2.0	0.7–5.5	0.187	0.1	0.1–0.3	< 0.0001^*^
**Infant testing coverage**						
No	***ref.***			***ref.***		
Yes	0.6	0.2–1.7	0.356	1.5	0.4–5.5	0.571
No answer/Don’t know	0.5	0.2–1.0	0.060	1.0	0.4–2.3	0.992
**Timing of First HIV positive result**						
Before most recent pregnancy	***ref.***			***ref.***		
At ANC first visit or after	1.1	0.7–1.7	0.716	1.2	0.7–2.2	0.465
**Experienced clinic visit barriers**						
No	***ref.***			***ref.***		
Yes	0.8	0.5–1.4	0.455	0.6	0.3 -1.0	0.048^*^

aRRR = Adjusted relative risk ratio; CI = confidence interval; Periurban_EC = OR Tambo District in Eastern Cape Province; Urban_GP = Ekurhuleni District in Gauteng Province; Urban_KZN = eThekwini District in KwaZulu-Natal Province; Rural/periurban_MP = Ehlanzeni District in Mpumalanga Province; Rural_LP = Greater Sekhukhune District in Limpopo Province; Rural_NW = Bojanala District in North West Province; SES = socio-economic status; *significant p-values at p < 0.05

Similar results were observed amongst the HIV-positive women sub-sample. Those who reported to be single and those who had an unplanned pregnancy were significantly more likely to not know their MPHIVs, aRRR 2.0; 95%CI 1.0-3.9; p = 0.038 and aRRR 2.4; 95%CI 1.4–4.3; *p* = 0.003, respectively. Those HIV-positive women who disclosed their HIV status to a partner and experienced clinic visit barriers were significantly less likely to not know their MPHIVs, aRRR 0.1; 95%CI 0.1–0.3; *p* < 0.0001 and aRRR 0.6; 95%CI 0.3-1.0; *p* = 0.048, respectively (Table 4).

### Observations from the qualitative data

#### Population characteristics

FGDs were successfully conducted amongst a mixed group of 37 pregnant women, 28 HIV-positive postpartum women, 34 HIV-negative postpartum women, 5 HIV-negative postpartum AGYW, 2 HIV-positive male partners and 7 HIV-negative male partners.

#### Emerging themes supporting quantiative results

Several themes emerged from the FGDs, some bringing emphasis to the observed quantitative results and others suggesting solutions to challenges related to the disclosure of MPHIVs.

### THEME 1: male partner’s reluctance to HIV testing

During FGDs, a theme on ‘*male partner’s reluctance to HIV testing’* emerged. Both women and male participants confirmed that male partners are very resistant to test for HIV so that they know their status. While some women expressed concerns that their partners secretly test for HIV and do not disclose their HIV status, others pointed out that some male partners preferred not to test but use the woman’s HIV results to assume their own HIV status. In some cases, discussions around the need for a male partner to test for HIV resulted in violence:

*“…and at times you might find he secretly tests at work and does not tell you and take the treatment without telling you…” Post-natal HIV-negative mother Mpumalanga*.

*“We fight often, …. Even if you offer to bring test kits from the clinic, so that you test together at home, he is not prepared to get tested, he will say you will get tested alone, he is not part of that arrangement.” Post-natal HIV-negative mother Eastern Cape*.

*“ …. one of the key things is that if a woman got tested it means I am also fine and that suits me to ensure that the baby is born HIV-negative…So it is also my responsibility to ensure that I don’t fool around and infect her with AIDS…or STIs… Because we men are scared to visit the clinic for testing, so if a woman is tested I know that I am also negative.” HIV-negative male partner Mpumalanga*.

*“He had no idea that I had to test but because he checks my card, he saw that I got tested and saw the results. He has never tested he just assumes that if I am okay then he is also okay. Their problem is that they are afraid to go to the clinics”. Pregnant HIV-negative mother KwaZulu Natal*.

Few men, however, mentioned their support for female partners to go for frequent couple testing.

*“…when I knew that she was pregnant, I did not have a problem with her being tested for HIV because that is what we have been doing. I was doing it even before I met her. I am the one who encouraged her to get tested since we met because she was still young when we started our relationship… I taught her about the importance of knowing her status, …Even now we often go for testing. I think we go for testing 3–4 times a year; our last test was last month.” HIV-negative male partner KwaZulu Natal*.

### THEME 2: increased difficulty in non-cohabiting male partner disclosure

A theme highlighting, ‘*increased difficulty in non-cohabiting male partner disclosure’* emerged from the FGDs, supporting the observed relationship between single marital status and not knowing a MPHIVs.

*“….… Disclosing to her was not easy at all, it was difficult, it took me some time to tell her*.***…****I think it took me about a year before I could tell her. I was hiding it from her all this time… Yes, I was using condoms… she was angry, and she asked why I did not tell her all this time?… yes, I was taking ARVs but I was hiding from her… We were not living together… I couldn’t stick to the same time, when I knew that she was visiting, I would take them before she arrives so that she does not see me taking them. Then I ended up deciding to tell her because I could see that this was problematic……” HIV-positive male partner KwaZulu Natal*.

One woman related about a relative who had multiple non-cohabiting sexual partners.

*“She [cousin] died when she was 43. She had different partners. She didn’t have a stable partner. Yes, she disclosed [her status] to her partners….One man (whom I knew) didn’t tell her that his wife had died before she dated him…That man already had the virus, but he didn’t tell her.” Postnatal HIV Negative mother North-West*.

### THEME 3: female partner initiating disclosure of HIV status

A FGD theme on ‘*female partner initiating disclosure of HIV status’*, emerged with mixed views and experiences some of which were positive and strongly supported the observed reduced risk of not knowing a MPHIVs when a woman has disclosed their own status to them. In these cases, woman’s initiation of disclosure influenced the male partner to also take an HIV test.

*“I told him****[****my results??] that he should also get tested; then he said OK it does not matter. He got tested and is also taking treatment…since 2017…he is supportive” Post-natal HIV-positive mother Mpumalanga*.

*“I have been always aware of my HIV status, I was testing regularly. But when I met my partner, she was honest enough with me. She told me that she is HIV-positive but because I love her I accepted her the way she is and we lived together, doing things together including taking care of me during difficult times in my life. We decided that we are going to use condom all the time but I am the one who did not protect myself because when I am drunk I would not use it, then I would go for testing and I would still be negative several times and she has been the one who would encourage me to go for testing. Then I ended up testing positive, but I did not stress her and even now, I don’t fight with her, I don’t criticize her because I know, I am the one who caused this situation…” HIV-positive male partner KwaZulu Natal*.

Other participants however, raised challenges related to disclosure of a positive status to male partners such as violence, stigma and blame, and lack of support.

*“When they don’t kill you, they walk away, and he assumes the disease came with you and not him. We don’t go to taverns and sleep around; and at times you might find he secretly tests at work and does not tell you and take the treatment without telling you. You will test and disclose to him and only then he will disclose and say he was scared to tell you*.***” ****Post-natal HIV-negative mother Mpumalanga*.

*”I think another challenge is when the mother of the baby is the first one to know about her HIV-positive status, then get blamed by her partner for transmitting the disease, disrespecting the woman in the process…” HIV-negative male partner KwaZulu Natal*.

*“Very few partners are supportive, sometimes when you disclose to them, they will say you are the one who infected him with the disease, then he does not bother to give you support.” Pregnant HIV-negative mother Mpumalanga*.

### THEME 4: Ways to support disclosure of male partner’s HIV status

Two sub-themes suggesting ways to support disclosure P-VHT users and their male partners arose from the FGDs and were (i) *healthcare worker support and (ii) community-based engagements*.

*Sub-theme on Healthcare worker support* included healthcare workers mediating the process of HIV disclosure between a P-VHT user and their male partner. This was achieved by either healthcare workers directly inviting male partners to attend P-VHT services or the female partners would insinuate the invitation as a healthcare worker’s directive.

*“.… I don’t know but I told the sisters at the clinic and they said they will call him so that he can come and check. He came but tested negative the first time and positive the second time. And they explained to him that he must come back in 3 months to test again. They told him to support me….” Post-natal HIV-positive mother Gauteng*.

*“My kid’s father also knows that when I come to the clinic I have to test and the reason he knows is because when I still had a little child I had to test often but I always told him that I was asked to come with him on my next visit so that he can test. I had to lie to him just to get him to check.” Pregnant HIV-negative mother KwaZulu Natal*.

*Sub-theme on community-based engagement* activities included door-to-door campaigns for HIV testing that subsequently promoted partner disclosure, and promotion of condom use in various community settings to prevent horizontal HIV transmission and unplanned pregnancies:

*“…. He got tested from the door-to-door campaign and I tested here at the clinic and we shared our results. He was happy that we are both negative and were happy that the child will be negative. We decided to use condoms going forward, we use protection.” Post-natal HIV-negative adolescent mother KwaZulu Natal*.

*“… I think condom use should be promoted mostly from schools, churches, malls because initially people were not using condoms, hence using condoms consistently is becoming a challenge. I also want to advise my fellows to carry condoms in their pockets everywhere they go. Whether you are going to work, for partying, condoms should be your friends until you get used to it.” HIV -negative male partner KwaZulu Natal*.

### THEME 5: evidence of high-risk sexual practices

Two sub-themes indicating evidence of high-risk sexual practices thus emphasizing on the need for partner HIV status disclosure, were also evident amongst non-cohabiting women. These women expressed concerns about male partners (i) “*having multiple sexual partners*” and at the same time being (ii) “*reluctant to use condoms*”.

Sub-theme- ‘male partners having multiple sexual partners’ emerged as follows:

*“I feel I am sometimes at risk. I do not know my partner’s status and I do not know where he is right now or what he’s getting up to; when he comes to visit me, we do not use condoms and that means I may get it.” Post-natal HIV-negative mother Eastern Cape*.

*“Men are troublesome, and they are selfish, because one knows that he has many relationships outside your relationship. When he is visiting you, he should protect you, but he does not want to use a condom. Most men are inconsiderate when it comes to their families.” Post-natal HIV-negative mother Mpumalanga*.

*“We as women have limits. So, you will get married and stay at home but our men will be running all over to collect other women. We don’t know them, so the risk is too high. I can tell myself that I’m protecting myself with one partner; I don’t see other men but him, he thinks he is the boss he can do whatever he wants.” Pregnant woman North-West*.

*Sub-theme- ‘Reluctance to use condoms’* by male partners was a very common theme in all FGDs and it supported the association observed between unplanned pregnancy and increased odds of not knowing a MPHIVs. Both male and female participants cited male partners frequently refusing to use condoms due to a variety of reasons:

*“Yes, I think condom is important, but I am also not using it most of the time. I also experience pain and also feel like it’s going to remain inside my partner.” HIV-negative male partner KwaZulu Natal*.

*“Men do not like condoms, they do not like using a condom. They refuse to use a condom and tell you that they do not like using a condom. They want flesh. Because he does not feel pleasure when he is using a condom…He wants to enjoy me well.” Post-natal HIV-negative mother Mpumalanga*.

*“Hey I am struggling with using condom, I manage to wear it and I do use it but after sexual intercourse I experience pain in my penis, hey excruciating pain. I don’t feel that pain if I did not use it…but I once ended up not using it with someone I was cheating with. I came to the clinic for testing and I was so hurt that I cheated on my partner such that I disclosed to her that I cheated on her without using condom…I am not promoting sex without condom, but I am speaking from the heart, I personally do not use condom with my partner, and I would never ever cheat on my partner…” HIV-negative male partner KwaZulu Natal*.

*“They [male partners] will complain about condoms: this thing is not fitting, the oil is causing [discomfort]…Ja, the oil from the condom is bad. They will ask: ‘Why are you telling me to use this now? Ok, that means you are not going to be my wife; so, I can go out and start looking for another. If you want to be good wife, that means you won’t make me use condoms because you trust me, and I trust you’. If you are weak you will agree with him on everything which is wrong.” Pregnant woman North-West*.

In some cases, partners who agreed to frequently test for HIV opted not to use condoms leading to unplanned pregnancies.

*“No, for me it was not a problem because testing was the first thing we did when we started our relationship although we did not plan to have a baby. She got tested again during pregnancy ….So she told me about her status and it was not a problem…But we continue testing our status to make sure that the baby remains healthy. She goes whenever she feels and I also go for testing whenever I feel like. I don’t want to lie, we never went for testing together.” HIV-negative male partner KwaZulu Natal*.

## Discussion

It is important to understand patterns of male partner disclosure to their pregnant and postpartum female partners to improve progress towards the P-VHT global targets. This study aimed to explore the prevalence of not knowing a MPHIVs and its associated factors amongst postpartum women in six districts with a high burden of antenatal HIV in South Africa. In the quantitative analysis we found that not knowing a MPHIVs is prevalent in over a quarter of P-VHT users and is associated with single marital status, unplanned pregnancy and woman not disclosing her own HIV status. Supporting studies have also shown that these factors have been associated with key P-VHT outcomes for many years [12, 31, 32]. The prevalence of not knowing a MPHIVs (28.7%) remains consistent with previous studies conducted in similar settings, examples include South Africa (34.0%) [33], India (33.0%) [34] and Malawi (26.8%) [4]. Our study findings are also consistent with those from studies reporting factors influencing disclosure in HIV-positive people as well as PPW to their partners. In studies assessing disclosure to sexual partners between men and women, identified factors also included marital status and knowledge of partner status [35–38].

The four themes which emerged from the FGDs of lived experiences of P-VHT users and their male partners, i.e., *male partner’s reluctance to present for HIV testing, increased difficulty in non-cohabiting male partner disclosure, high-risk sexual practices (with reluctance to use condoms and multiple sexual partners as sub-themes) and female partner initiating disclosure of HIV status’, all* confirmed the observed statistical associations and enhanced our understanding of the underlying challenges and opportunities for improvement. Two additional sub-themes for supporting disclosure, *healthcare worker support* and *community-based engagements*, provided other user-informed opportunities to improve male partner’s disclosure of HIV status. Qualitative findings suggested that disclosure of HIV status by male partners to their pregnant or postpartum partners was associated with planned pregnancy among the couples. Other studies have also found that gender [35], timing of knowledge of HIV status and age [36, 39] were factors associated with disclosure to male partners in PPW, while being on ART was a factor for disclosure among sexual partners and postpartum women to their male partners [37, 38]. We found that many participants who reported having disclosed to partners, reported engaging in safer sex and receiving support. This is consistent with previous studies showing that disclosure to partners increases the likelihood of regular condom use with partners [18, 37, 40]. Others however, feared being stigmatized or blamed by their partners which could also lead to withdrawal of partner support and/or violence. These results relate to other studies that reported that disclosure of HIV status by partners was associated with less perceived stigma and lack of anticipated outcome such as rejection and blame [35, 38, 41–43]. Other factors significantly associated with increased partner disclosure included receiving pre-and- post- test counselling, being Christian versus Muslim, having discussed testing with partner prior and having a good relationship with partner [35, 36, 39, 44].

Therefore, these results confirm that not knowing a MPHIVs needs to be addressed urgently in order to improve progress towards achieving the P-VHT targets. The mixed method approach of our study has contributed both qualitative and quantitative factors of not knowing MPHIVs and in the qualitative analysis the authors were able to identify other factors that were not included in the quantitative analysis variables. The qualitative analysis also indicated important future interventions for support in HIV disclosure. Additionally, this study explored factors from both the perspectives of the male partner as well as the PPW while previous studies only reported the perspectives of the women.

The reluctance to test for HIV amongst men is a global problem that exists beyond the P-VHT context. Globally, there are 8% more men with unknown HIV status, 11% more men not on ART and 10% more men not virally suppressed compared to their women counterparts [45]. Research from various regions in South Africa including Gauteng, Mpumalanga and KZN has shown that unknown HIV status and unprotective sex are risk factors for HIV transmission and other STIs [11, 22, 46–48] and need to be addressed by introducing multifaceted interventions within and outside of the P-VHT programme. Within the P-VHT context, various studies have reported that knowing a MPHIVs resulted in improved clinical outcomes, increased partner support, increased ART uptake and adherence [4, 5, 7, 8]. Although the descriptive results in our study showed that most of the women who faced challenges with ART adherence were in known HIV-positive concordant relationships, the magnitude of the odds did not indicate any significant associations between a woman’s knowledge of MPHIVs and ART challenges. A larger sample size could be required to confirm whether there is an underlying relationship between known male partner HIV status and ART uptake indicators in this population. However, the findings of our study emphasize on the urgency to address male partners’ resistance to HIV testing or disclosure of HIV-positive status and refusal or inconsistent use of condoms.

Interventions to increase male partner involvement in P-VHT including HIV testing and disclosure are important for the success of P-VHT programmes [49] and reaching the global targets. Pillar 4 of the 2019 South African National P-VHT Guidelines includes “providing appropriate treatment, care, and support to women, their children, partners and families” [9], this indicates that policy makers are aware of the role male partners play in P-VHT programmes. Implementation of this policy should be strengthened by combination programmes aiming to enhance P-VHT dialogue and involvement in male partners of PPW including uptake in HIV prevention and care services [49]. Engaging male partners in P-VHT programmes as either clients, partners or as agents advocating for improved knowledge in P-VHT, health seeking behaviour, de-stigmatization of HIV and gender-based violence through dialogue and sensitization via radio stations or home visits of both male partners and women, has been effective in increasing testing and male involvement [50–56].

Structural interventions have also been implemented and these include shorter waiting times for pregnant women attending the clinic with their partners and extended clinic hours for male partners to minimize the barrier of work commitments [50, 57]. Structural barriers, however, have included lack of space in antenatal clinics to accommodate male partners, which will need to be addressed if male participation continues to be encouraged. Programmes in South Africa and Nigeria which include various combinations of the above-mentioned interventions have resulted in an increased uptake of P-VHT programmes in both pregnant women and their male partners including HIV testing, disclosure and improved P-VHT outcomes [58–61]. However, there is room for more work to be done in the P-VHT context, given the observed high prevalence of not knowing a MPHIVs. In line with this, qualitative discussants in this study highlighted the need for strengthening HCWs’ support in involving and including their male partners. HCW assisted disclosure might also reduce the risk of abuse, rejection, and stigma against the disclosing partner.

In addition to HCW-supported disclosure, another theme arising from this study’s FGDs was the strengthening of community-based interventions. Community-based peer education and support interventions that would promote condom use, HIV testing and male involvement were highlighted. Some initiatives already exist such as a ‘door-to-door’ campaign through which some discussants had the opportunity to test and disclose their HIV status to one another. Several other home-based interventions such as the distribution of HIV test-kits, couple testing, partner notification services and education have increased male partner testing in some low-middle income countries [62–70]. Providing integrated care (couple testing, one-stop shop for couples and families) is another possible intervention which can strengthen existing effort to eliminate barriers to inter-partner disclosure. Community based strategies should also strengthen initiatives for training counsellors to conduct on-going counselling and support to partners, emphasizing on positive outcomes of disclosure and address disclosure barriers. Clients should be empowered in the process, about how to deal with negative outcomes of disclosing a HIV-positive result.

The community environment is also ideal for addressing gender norms which impact negatively on women’s sexual and HIV health. Most women interviewed were aware of their risk of exposure to HIV infection and some were even willing to engage in safe sexual practices, however their male partners were dominant in the decision making for condom use. Findings from this study resonate with other studies which reported on how gender inequalities, societal and cultural norms influence vulnerability of women to HIV infection [71–73]. This therefore implies that HIV prevention interventions should also address gender inequities and include all the sectors of the society including youth, religious and traditional organisations as well as health, education and social welfare departments. A community mobilization intervention which aimed at challenging gender norms, reducing HIV stigma and fear of disclosure improved testing uptake and prevented men from relying on their partner’s results [74].

It was apparent that male partners are dominant in deciding condom use. In addition to promoting pre-exposure prophylaxis (PrEP), and due to the concerns about male partners’ hesitancy to use condoms consistently and likelihood of multiple sexual partners especially when not co-habiting with primary partner, P-VHT HTSs approach might increase awareness and accessibility of the complementary female condom as an alternative to empower women to protect themselves. The normalization of the female condom is also likely to reduce the rate of unplanned pregnancies. Very few participants reported condom use after knowing their HIV status, and even amongst those, consistent condom use was reported as a challenge. Common challenges that were reported for not using condoms consistently included hating the smell of the condom lubricant, skin reactions, pain and discomfort for the male partners, reduced pleasure and being under the influence of alcohol. Some males reported consistent condom use during the first 3 months into the relationship and thereafter, stopped regardless of whether partners disclosed HIV status or not, as perceived sign of loyalty. Female partners were often made to feel guilty for insisting on condom use. Their male partners interpreted this as an indicator of promiscuity.

Positive attitudes towards PrEP were observed from the discussants. Given the low disclosure of male HIV status and non-condom use in South Africa, many women felt it would be necessary to make PrEP readily available at the clinics to women to either choose to use it consistently or during their time of exposure to HIV risk (interrupted). Those who proposed interrupted use cited fear of disclosing the use of PrEP to their male partners. Women who did not perceive challenges in the disclosure of PrEP use were willing to take it for a lifetime or for the period of being involved in a sexual relationship. Few participants felt that men should be encouraged to take PrEP since it was perceived that it is the male partners who engage in multiple sexual relationships. The dissemination of PrEP therefore, needs to put men at the forefront to lead the peer education initiatives and promotion of PrEP in communities.

### Limitations and strengths

The results from the quantitative component may not be generalizable to the broader population due to selection bias from recruiting postpartum women attending healthcare centers only and not including male partners. Furthermore, the men included in the qualitative study were only included in the study if they were attending the maternity or other mother and child health services resulting in fewer participating men, recruited from only two districts, compared to women. Only one AGYW participated in the qualitative study. Views of the participants from other districts may not be the same as those that participated in the study. The HIV status of some male partners and pregnant women was not confirmed. Despite these limitations, the strengths of this study included both women and male partners from high burden HIV areas, as well as a complementary mixed methods design.

## Conclusion and recommendations

Innovative efforts are needed to close the gap of MPHIVs non-disclosure amongst women of child-bearing age, particularly those at risk of unstable sexual partners and unplanned pregnancies. FDGs highlighted some complexities around MPHIVs disclosure and provided clues for designing and strengthening user-informed and potentially effective interventions to close this gap. Several interventions have already been tested in various settings. However, there is clear evidence for the need to strengthen community-based interventions and to broaden the impact of the current P-VHT service delivery policy to reach male partners, routinely educate women and men about safer sexual practices and empower them to take advantage of other prevention alternatives such as PrEP and condoms.

### Electronic supplementary material

Below is the link to the electronic supplementary material.

Additional file 1: COREQ Checklist for Qualitative Study

Additional file 2: Option B+ FGDs inclusion criteria

## Data Availability

The dataset is still being analyzed by the primary research team. Anyone needing to access the data should e-mail Ameena.Goga@mrc.ac.za for quantitative data or Vuyolwethu.Magasana@mrc.ac.za for qualitative data and qualitative methodology. Any data sharing will be by individual request, and in consultation with researchers currently analyzing the data.

## List of abbreviations

ART: antiretroviral therapy
COREQ: Consolidated criteria for Reporting Qualitative research
HIV: Human immunodeficiency virus
HTSs: HIV testing services
MPHIVs: Male partner’s HIV status
VHT: vertical Human immunodeficiency virus transmission
P-VHT: Prevention of vertical Human immunodeficiency virus transmission
