# Supplementary material for: Mixed-methods cross-sectional study of the prevention of vertical HIV transmission program users unaware of male partner’s HIV status, in six South African districts with a high antenatal HIV burden
Source: BMC Public Health. 2023 Oct 12;23:1988. doi: 10.1186/s12889-023-16921-z (PMC10571358; doi:10.1186/s12889-023-16921-z)
Supplement: Supplementary file 1 — Additional file 1: COREQ Checklist for Qualitative Study [file 12889_2023_16921_MOESM1_ESM.docx]

**Additional file 1: COREQ Checklist for Qualitative Study**

**Domain 1: Research team and reflexivity**

**Personal Characteristics**

*1. Interviewer/facilitator - Which author/s conducted the interview or focus group? (Page 28).*

- VM
- DFN

*2. Credentials - What were the researcher’s credentials? (Page 8 and 9).*

- VM (MPH)
- DFN (MPH)
- MG (PhD candidate) who supported during data analysis

*3. Occupation - What was their occupation at the time of the study? (Page 8 and 9).*

- VM - Project Manager, Health Systems Research Unit, South African Medical Research Council
- DFN - Project Leader, Health Systems Research Unit, South African Medical Research Council
- MG - A private consultant- secondary analyst.

*4. Gender - Was the researcher male or female? (Page 8)*

- All researchers (data collection, coding/data analysis) were female

*5. Experience and training - What experience or training did the researcher have?*

- VM- Completed MPH. She had performed numerous qualitative and mixed method investigations and has more than 18 years’ experience in maternal and child health research.
- DFN – is a registered nurse -midwife with a Hons degree in nursing education and MPH degree. She has conducted many qualitative and mixed methods research projects and has more than 20 years’ experience in maternal and child health research.
- MG- a qualitative researcher with 14 years’ experience in collecting and analysing qualitative research data

*6. Relationship with participants. Was a relationship established prior to study commencement? (Page 8)*

- No. Both researchers had no previous relationship or contact with participants.

*7. Participant knowledge of the interviewer - What did the participants know about the researcher?*

- Participants did not have prior knowledge about researchers before FGDs.

*8. Interviewer characteristics - What characteristics were reported about the interviewer/facilitator?*

- Information about the interviewers’ employer was given to participants during the informed consent process.

**Domain 2: Study design**

**Pre-structured qualitative research design**

*9. Methodological orientation and Theory - What methodological orientation was stated to underpin the*

*study? (Page 9)*

- Inductive approach

**Participant selection**

*10. Sampling - How were participants selected? (Page 8)*

- Purposive sampling method was used.

*11. Method of approach - How were participants approached?*

- Participants were addressed initially as a group by the researchers about the purpose of the study while waiting to be attended in the clinic then approached individually to administer informed consent.

*12. Sample size - How many participants were in the study? (Page 10)*

- 113 participants participated in FGDs.

*13. Non-participation- How many people refused to participate or dropped out? Reasons?*

- These data were not collected.

**Setting**

*14. Setting of data collection - Where was the data collected? (Page 8)*

- In primary health care facilities within purposively selected districts, using face-to-face methods.

15. *Presence of non-participants. Was anyone else present besides the participants and researchers?*

- No

*16. Description of sample - What are the important characteristics of the sample? (Page 8, 12 and additional file 2)*

- Pregnant women, HIV-positive postpartum women, HIV-negative postpartum women, HIV-negative postpartum adolescent women, HIV-positive male partners and HIV-negative male partners

**Data collection**

*17. Interview guide - Were questions, prompts, guides provided by the authors? Was it pilot tested? (Page8)*

- Interview guides with probes were provided but not pilot tested prior to use in this study.

*18. Repeat interviews - Were repeat interviews carried out?*

- No.

*19. Audio/visual recording - Did the research use audio or visual recording to collect the data? (Page 8 and 9)*

- Interviews were audio recorded and transcribed verbatim.

*20. Field notes - Were field notes made during and/or after the interview or focus group? (Page 8)*

- Yes, field notes were taken during the interviews.

*21. Duration - What was the duration of the interviews or focus group? (Page 8)*

- Interviews lasted approximately 45 minutes.

*22. Data saturation - Was data saturation discussed? (Page 8)*

- Yes

*23. Transcripts returned - Were transcripts returned to participants for comment and/or correction?*

- No, the transcripts were not returned to the participants. Researchers checked for accuracy of transcriptions of audio recordings prior to data analysis.

**Domain 3: Analysis and findings**

**Data analysis**

*24. Number of data coders - How many data coders coded the data? (Page 9)*

- Three researchers coded the first 10 interviews (VM, DFN, MG), then two researchers coded the remaining interviews.

25. Description of the coding tree - Did authors provide a description of the coding tree?

- No

*26. Derivation of themes - Were themes identified in advance or derived from the data? (Page 9)*

- Inductive – Themes were derived from the data

*27. Software - What software, if applicable, was used to manage the data?*

- No software was used

*28. Participant checking - Did participants provide feedback on the findings?*

- No

**Reporting**

*29. Quotations presented - Were participant quotations presented to illustrate the themes / findings? Was each quotation identified?*

- Yes, quotations were presented to illustrate the themes identified. Each quotation was identified only by the type of FGD and the district where it was conducted to endure confidentiality.

*30. Data and findings consistent - Was there consistency between the data presented and the findings?*

- Yes, the quotes relate to the themes identified.

*31. Clarity of major themes - Were major themes clearly presented in the findings?*

- Yes, the major themes are presented throughout the results section including illustrative quotes.

*32. Clarity of minor themes - Is there a description of diverse cases or discussion of minor themes?*

- Yes, main themes have been provided and we discussed diverse cases in the results.
