# Supplementary material for: Mixed-methods cross-sectional study of the prevention of vertical HIV transmission program users unaware of male partner’s HIV status, in six South African districts with a high antenatal HIV burden
Source: BMC Public Health. 2023 Oct 12;23:1988. doi: 10.1186/s12889-023-16921-z (PMC10571358; doi:10.1186/s12889-023-16921-z)
Supplement: Supplementary file 2 — Additional file 2: Option B+ FGDs inclusion criteria [file 12889_2023_16921_MOESM2_ESM.docx]

**Additional file 2: Option B+ FGDs inclusion criteria**

- Group 1: 8-10 HIV positive mothers who are 25 years old and above with babies aged 0-6 months old who use health services in the district and have lived in this district for more than 6 months
- Group 2: 8-10 HIV positive mothers who are less than 20 years old with babies aged 0-6 months old who use health services in district and have lived in this district for more than 6 months
- Group 3: 8-10 pregnant women (HIV positive and negative) who use health services in district and have lived in this district for more than 6 months, 2 aged <20 years; 2 aged 20-24 years, 2 aged 25-29 years, 2 aged 30-34 years, 2 aged >35 years
- Group 4: 8-10 men with children younger than 6 months who use health services in district and have lived in this district for more than 6 months
- Group 5: 8-10 HIV negative mothers (with documented HIV negative test result within the last 3 months) who are 25 years old and above with babies aged 0-6 months old who use health services in district and have lived in this district for more than 6 months
- Group 6: 8-10 HIV negative mothers (with documented HIV negative test result within the last 3 months) who are less than 20 years old with babies aged 0-6 months old
